# 

**Published:** 1920-01-03

**Authors:** 


### THE HOSPITAL

### The Modern Newspaper of Administrative Medicine and Institutional Life.

Telegraphic Address: OSPEDALE, RAND, LONDON. Telephone Number: 2734 GERRARD

No. 1752, Vol. LXVII. | Saturday, January 3rd, 1920. | PRICE TWOPENCE

